# Meat Quality and Muscle Tissue Proteome of Crossbred Bulls Finished under Feedlot Using Wet Distiller Grains By-Product

**DOI:** 10.3390/foods11203233

**Published:** 2022-10-17

**Authors:** Welder Baldassini, Mohammed Gagaoua, Bismarck Santiago, Leone Rocha, Juliana Torrecilhas, Rodrigo Torres, Rogério Curi, Otávio Machado Neto, Pedro Padilha, Felipe Santos, Dante Pazzanese Lanna, Luis Artur Chardulo

**Affiliations:** 1 School of Veterinary Medicine and Animal Science (FMVZ), São Paulo State University (UNESP), Botucatu 18618-681, São Paulo, Brazil; 2 School of Agriculture and Veterinary Sciences (FCAV), São Paulo State University (UNESP), Jaboticabal 14884-900, São Paulo, Brazil; 3 Food Quality and Sensory Science Department, Teagasc Food Research Centre, Ashtown, Dublin 15, D15 DY05 Dublin, Ireland; 4 PEGASE, INRAE, Institut Agro, 35590 Saint-Gilles, France; 5 Institute of Bioscience (IB), São Paulo State University (UNESP), Botucatu 18618-689, São Paulo, Brazil; 6 College of Sciences and Engineering, São Paulo State University (UNESP), Tupã 17602-496, São Paulo, Brazil; 7 “Luiz de Queiroz” College of Agriculture (ESALQ), University of São Paulo (USP), Piracicaba 13418-900, São Paulo, Brazil

**Keywords:** beef quality, cattle, feedlot, proteome, mass spectrometry, nutrigenomics, skeletal muscle

## Abstract

Wet distiller grains (WDG) are a corn by-product rich in protein and fiber that can be used in feedlot diets. This study evaluated F1 Angus-Nellore bulls fed on a control diet vs. WDG (*n* = 25/treatment). After a period of 129 days on these feeds, the animals were slaughtered and *Longissimus* *thoracis* samples were collected for both a meat quality evaluation and gel-based proteomic analyses. A greater ribeye area (99.47 cm²) and higher carcass weight (333.6 kg) (*p* < 0.05) were observed in the WDG-finished cattle compared to the control (80.7 cm²; 306.3 kg). Furthermore, there were differences (*p* < 0.05) in the intramuscular fat between the WDG and control animals (IMF = 2.77 vs. 4.19%), which led to a significant decrease (*p* < 0.05) in saturated fatty acids (FA). However, no differences (*p* > 0.10) were observed in terms of tenderness, evaluated using Warner–Bratzler shear force (WBSF). The proteomic and bioinformatic analyses revealed substantial changes in the biological processes, molecular functions, and cellular components of the WDG-finished cattle compared to the control. Proteins related to a myriad of interconnected pathways, such as contractile and structural pathways, energy metabolism, oxidative stress and cell redox homeostasis, and transport and signaling. In this experiment, the use of WDG supplementation influenced the protein expression of several proteins, some of which are known biomarkers of beef quality (tenderness and color), as well as the protein–protein interactions that can act as the origins of increases in muscle growth and reductions in IMF deposition. However, despite the effects on the proteome, the tenderness, evaluated by WBSF, and fatty acid profile were not compromised by WDG supplementation.

## 1. Introduction

Wet corn distiller grains (WDG) have been extensively used for cattle feeding, especially in the USA, for more than 30 years [1]. The inclusion of WDG in feedlot diets is used strategically by feedlot owners who seek ingredients from the agroindustry in an attempt to reduce costs [2,3]. In the mid-west region of Brazil, where ethanol is produced from corn, the number of feedlot owners using this ingredient for feedlot cattle has been increasing.

Replacing corn/soybean meal ingredients with distiller grains can lead to a reduction in dietary starch levels, as well as changes in dietary protein degradability [4]. Corn WDG is an ingredient rich in rumen undegradable protein, and an earlier study [5] suggested that an increased protein supply to the intestine may stimulate pancreatic amylase secretion, hence increasing post-ruminal starch digestion. In this context, the use of distiller grains as an energy source in cattle feedlots represents a major paradigm shift, since these grains a protein feed [6].

Earlier studies evaluated the effects of high-concentrate (80%) diets containing increasing levels of WDG (0%, 30% or 60%) in Simmental-Angus steers [7]. The authors observed that traits such as the carcass yield, hot carcass weight, and subcutaneous fat thickness responded positively to the inclusion of 30% WDG, while the inclusion of 60% had a negative impact on these carcass traits. Similarly, a study of the effect of the inclusion of 15% dried distiller grains with solubles (DDGS) in the feedlot diet on the carcass finishing traits and beef lipid profile in Holstein steers was reported [8]. The study reported no difference in the quantity of subcutaneous fat between animals that received DDGS and the control. The fatty acid profile of the beef was also not affected. However, these authors did not describe the effects of distiller grains on the molecular changes at the muscle proteome level [7,8] and its association with the carcass and meat quality traits.

Studies have investigated the molecular alterations associated with beef quality traits such as tenderness and marbling [9,10]. However, few have evaluated the biochemical and molecular mechanisms that regulate the tenderness and marbling of meat from cattle fed a feedlot diet containing WDG. Such an evaluation of the bovine muscle tissue proteome is of great importance for the meat industry in order to add value to the end product [11,12]. Furthermore, there are a limited number of proteomic studies involving feedlot-finished, non-castrated crossbred males [13,14]. This biological type is increasingly being used in the tropical regions of Brazil, especially for F1 Angus-Nellore bulls, whose producers seek to satisfy more demanding consumer markets that require products with greater added value in terms of their sensory traits, especially meat tenderness and juiciness. Therefore, there is a growing expectation placed on the meatpacking industry regarding this genotype for the production of higher quality meat, as well as the use of by-products such as WDG by the producers of feedlot cattle. With the objective of better understanding the impacts of such supplementations and feeding methods on meat quality variation and muscle proteome changes, this study aimed to evaluate the meat quality and muscle tissue proteome of crossbred cattle fed on a feedlot diet containing WDG.

## 2. Materials and Methods

The animal study protocol was approved by Ethics Committee (CEUA) of the São Paulo State University “Júlio de Mesquita Filho”, UNESP, Campus of Botucatu (protocol number 0067/2017).

In this trial, F1 Angus-Nellore bulls (*n* = 50) from a commercial herd with an average initial body weight of 369.58 ± 49.17 kg, aged 20–24 months, were used. The animals were kept in collective pens (5 × 6 m, capacity of five animals per pen) with concrete a floor, equipped with a shell-type water trough. The pens were randomly allocated one of two treatments (25 animals/treatment), which consisted of a control (0%) vs. low-fat corn WDG (45%, DM basis). Feedlot diets (Table S1) include roughage (Tifton hay and sugarcane bagasse) and concentrate ingredients (ground dry corn grain, soybean meal, corn WDG, mineral core). The low-fat WDG used in the current study was produced by the same ethanol industry (SJC Bioenergia, Quirinópolis, Goiás, Brazil). The animals received the experimental diets ad libitum twice a day (10 a.m. and 4 p.m.) for 129 days and reached an average final body weight of 615.09 ± 57.53 kg.

### 2.1. Slaughtering, Sampling, and Carcass Quality Trait Evaluation

The animals were stunned by brain concussion with a captive dart pistol, followed by bleeding, hide removal, and evisceration. Subsequently, the carcasses were divided longitudinally, and samples (approximately 10 g) of the *Longissimus thoracis* (LT) muscle were collected from the left half of the carcass (approximately 10 min after slaughter) and stored using liquid nitrogen. These samples were then stored at −80 °C until molecular the biology and proteomic analysis.

The carcasses were weighed and cooled at 2 to 4 °C for 48 h. The hot carcass weight (HCW), cold carcass weight (CCW), backfat thickness (BFT, mm), and ribeye area (REA, cm^2^) at the 12th/13th rib interface were measured. Next, meat samples were collected from the LT muscle during deboning for the meat trait evaluation after three aging periods. All meat samples were collected between the 11th and 13th ribs (cranial direction).

### 2.2. Aging, Chemical Composition, and Marbling Score Determination of the Meat Samples

The steaks (2.54 cm thickness) were vacuum-packed in polyethylene bags and kept in a refrigerator for 3, 10, and 17 days post-mortem. Briefly, the samples were aged in a refrigerated BOD incubator (TE-371, TECNAL, Piracicaba, São Paulo, Brazil) at 0 to 2 °C in special plastic bags to ensure high vacuum conditions and a low oxygen permeability. The chemical composition was analyzed by infrared spectroscopy using a FoodScan^TM^ (Foss NIRSystems, Laurel, MD, USA). The intramuscular fat (IMF) content was determined following previous procedures [15]. Additionally, the IMF was quantified chemically using a gravimetric method, as described previously [16]. Visual marbling scores were determined by two trained panelists for each slice of steak, using official USDA marbling photographs (prepared by the National Cattlemen Beef Association) as a reference [17]. The marbling categories were 1 = devoid, 2 = practically devoid, 3 = traces, 4 = slight, 5 = small, 6 = modest, 7 = moderate, 8 = slightly abundant, and 9 = moderately abundant. However, in the current study, this scale ranged from zero (devoid) to six (modest) because of the low degree of marbling.

### 2.3. Shear Force, Cooking Loss, and Myofibrillar Proteolysis

The Warner–Bratzler shear force (WBSF) and cooking loss (CL) were measured following each aging time (3, 10, and 17 days post-mortem). The recommendations of the American Meat Science Association were followed [18]. The samples were placed on a grid coupled with a glass refractory. A thermocouple connected to a digital thermometer (DT-612, ATP Instrumentation, Ashby-de-la-Zouch, UK) was used, which was inserted into the center of each sample to monitor the internal end-point temperature.

The steaks were cooked in an industrial electric oven (Feri90, Venâncio Aires, Rio Grande do Sul, Brazil) at 170 °C. Once the internal temperature of the steaks reached 40 °C, they were turned over and remained in the oven until the final temperature reached 71 °C. Subsequently, the beef samples were then kept at room temperature for 15 min, weighed, and refrigerated at 4 °C for 24 h.

The CL was divided into the evaporation loss (EL) and drip loss (DL), determined as percentages. The DL was obtained by weighing, using only the refractory, before and after cooking the sample. The EL was obtained by weighing the sample before and after cooking.

For the determination of the WBSF, cylinders with a diameter of 1.27 cm were applied parallel to the muscle fiber, with a hollow punch coupled with an industrial drill. Eight cylinders of each sample were sheared (perpendicular to the fiber direction) using a Brookfield CT-3 Texture Analyzer (AMETEK Brookfield, Middleborough, MA, USA), equipped with a stainless steel 3.07 mm-thick Warner–Bratzler blade with a V-shaped (60° angle) cutting edge [19]. The results were reported as the average of eight values per sample in kilograms (kg).

Post-mortem proteolysis was estimated by determining the myofibril fragmentation index (MFI), as previously described [20], and adapted for *Bos indicus* cattle [21].

### 2.4. Fatty Acid Profile

Additional LT samples were used to extract IMF and were subsequently used for the fatty acid (FA) profile analysis by gas chromatography. The IMF was extracted according to the procedures described in [22], and the samples were converted into fatty acid esters, as described in [23]. The transmethylated samples were analyzed using a gas chromatograph (Focus GC-Finnigan, Thermo Finnigan, Milan, Italy) equipped with a flame ionization detector and a CP-Sil 88 capillary column (Varian; 100 m long, 0.25 µm internal diameter, and film thickness of 0.20 µm). Hydrogen was used as the carrier gas at a flow rate of 1.8 mL/min. The initial oven temperature program was 70 °C—holding time 4 min, 175 °C (13 °C/min)—waiting time 27 min, and 215 °C (4 °C/min)—waiting time 9 min, followed by increments of 7 °C/min up to 230 °C, retained for 5 min and totaling 65 min. The temperatures of the vaporizer and detector were 250 °C and 300 °C, respectively.

Aliquot (1.0 μL) of the esterified extract was injected into the chromatograph device. All FAs were identified by comparing the retention times of the methyl esters of the samples with the FA standards using the SupelcoTM Component Mix (cat. 18919; Supelco, Bellefonte, PA, USA) and rumenic acid c9, t11 CLA (cat. 05632; Sigma-Aldrich Corporation, St. Louis, MO, USA). The percentages of fatty acids were obtained using the Chromquest 4.1 software (Thermo Electron, Milan, Italy). The FA results were expressed as area percentages (feeds) and as mg/100 g of meat.

### 2.5. Proteome

The muscle tissue proteome of the animals was investigated by two-dimensional polyacrylamide gel electrophoresis (2D-PAGE) and electrospray ionization mass spectrometry (ESI-MS/MS), following procedures described in the literature [24,25].

#### 2.5.1. Protein Extraction and Precipitation

Individual biopsy samples of LT muscle collected early on post-mortem were used for protein extraction and separation on two-dimensional electrophoresis gels (2D-PAGE). For each treatment, ten individual samples (biological replicates) were used, with three technical replicates (three gels per sample). Approximately 0.2 g of each muscle sample was ground twice in 1.0 mL lysis buffer using an Ultra-Turrax high-shear mixer (Marconi—MA102/E, Piracicaba, São Paulo, Brazil) at 20,000 rpm for 30 s. The protein extracts were separated from the solid part by centrifugation at 10,000 rpm for 15 min at 4 °C. The protein contents of these extracts were stored in 80% (*v/v*) acetone solution in a refrigerator at 5 °C for 2–3 h to ensure that the procedure occurred for a sufficient period of time. The centrifugation process was then repeated for 25 min at 10,000 rpm to obtain protein pellets for quantification and 2D-PAGE.

#### 2.5.2. Protein Separation by Two-Dimensional Polyacrylamide Gel Electrophoresis

The total protein concentration of the bovine muscle tissue samples was quantified using the Biuret method [26]. The protein concentrations obtained were used to calculate the volume of the samples and the solution needed for electrophoresis, considering a protein mass of 375 μg and a volume of 250 μL applied to the strip, resulting in a total protein concentration of 1.5 μg/μL. The samples were solubilized in a solution containing urea, thiourea, (3-[(3-cholaminopropyl)-dimethylammonium]-1-propanesulfonate), ampholytes, bromophenol blue, and dithiothreitol. An aliquot of each sample of 250 μL was added to a 13 cm isoelectric focusing strip containing an immobilized ampholyte with a pH gradient from 3 to 10, which was subjected to hydration for 12 h.

After this period, the strips were subjected to the first step (1D) of two-dimensional electrophoresis, using an Ettan IPGphor 3 device (GE Healthcare, Chicago, IL, USA), in which the proteins were fractionated by the isoelectric point (pI), the pH value when the net charge total protein is zero. The strips were placed in equilibrium solutions for reduction and alkylation and subjected to the second stage (2D) of electrophoresis for the fractionation of the protein spots according to the molecular weight on a 12.5% polyacrylamide gel. At the end of the 2D run, approximately 500 mL of colloidal Coomassie stain was used to stain the protein spots of the gels. The gels were decolorized with ultrapure water after 72 h.

#### 2.5.3. Image Processing

The gels were scanned and the images were imported into the ImageMaster Platinum software for comparisons (contrasts) of the images between treatments and to obtain information such as the number of spots per gel, % matching (correspondence between the protein spots in the gels), isoelectric point (pI), molecular weight (MW), and spot volume. The gel correspondence (matching) of each sample (three technical replicates) was higher than 95%, i.e., 95% of the spots were present in the technical replicates, indicating a good reproducibility. For the image comparison, one reference gel per treatment was selected [27], which contained the largest number of most well-defined spots. The reference gel of the treatment was compared with each gel used for the other treatments.

#### 2.5.4. Tryptic Digestion of Protein Spots and Identification of Proteins by ESI-MS/MS

Protein spots from the experimental groups (control vs. WDG) were selected based on their MW and pI obtained by image analysis, cut out (fragments of approximately 1 mm^3^), and prepared according to the method described in [28]. In brief, the sediments were transferred to microtubes and submitted to the following four steps: (1) removal of the dye; (2) reduction and alkylation; (3) trypsin digestion (Trypsin Gold Mass Spectrometry, Promega, Madison, WI, USA); and (4) elution of peptides extracted from the gel. Subsequently, mass spectra of the peptides were obtained by analyzing the aliquots of the solutions using a nanoACQUITY UPLC-Xevo TQ-MS System (Waters, Manchester, UK). The proteins of the *Bos taurus* genome were identified in the UniProt database (UniProtKB/Swiss-Prot, available online: www.uniprot.org, accessed on 1 August 2022).

#### 2.5.5. Bioinformatics Procedures

Bioinformatic analyses were conducted for the classification of differentially expressed proteins in the muscle tissues from the animals of the control vs. WDG treatment group in terms of biological processes (BPs), the molecular function (MF), and cellular components (CC). For this purpose, the accession numbers of the proteins identified by ESI/MS/MS were entered into the UniProt database (available online: www.uniprot.org, accessed on 1 August 2022), and their FASTA sequences were extracted. After this step, the proteins were analyzed using the OMICSBOX v.2.0 (available online: https://www.biobam.com/omicsbox/, accessed on 1 August 2022) and Blast2GO tools [29].

Additionally, the interactions between the proteins identified in the treatments were analyzed using the open-source STRING 11.0 platform (available online: https://string-db.org/, accessed on 1 August 2022). The same list of proteins whose expression differed between the experimental groups were used in these analyses [30]. The minimum required interaction score was set at 0.900 (highest confidence), and no more than 20 interactions were allowed in the database search.

Subsequently, further bioinformatic analyses were performed, following the procedures described by Gagaoua et al. [31], using the Metascape^®^ platform. Briefly, gene identifiers were converted using Uniprot Retrieve/ID mapping. Thus, key information regarding the proteins (gene names (GN)) and its relationships with the carcass and meat quality traits described in the current study were annotated for each treatment (control and WDG). These procedures aimed to compare the two protein lists so as to better understand the common and divergent molecular signatures. Hierarchical heatmap clustering was also carried out using enriched GO terms analyzed by Metascape^®^ (available online: https://metascape.org/, accessed on 1 August 2022).

Additionally, the ProteQTL tool, included in ProteINSIDE (available online: http://www.proteinside.org/, accessed on 1 August 2022), was used for the rapid search of carcass and meat quality quantitative trait loci (QTL) among the list of putative biomarkers. ProteQTL interrogates a public library of published QTLs in Animal QTL Database (available online https://www.animalgenome.org/QTLdb, accessed on 1 August 2022) that contains cattle QTLs and association data collected from published scientific articles.

#### 2.5.6. Data Analysis

The data were submitted to analysis of variance (ANOVA) by the F test using the GLM procedure of the Statistical Analysis System (SAS, version 9.1, Cary, NC, USA). Means were compared by the Tukey test and a *p* value < 0.05 was adopted as the critical probability. The design was completely randomized, and the following model was used:

Yij = μ + ti + εij

where Yij is the observed value of the experimental unit referring to treatment i in repetition j; μ is the general effect of the mean; t is the treatment effect (diet); and ε is the experimental error.

Regarding the proteome data, the protein spot volume was imported into ImageMaster Platinum software and the mean and standard deviation were calculated for the selected spots. The images were compared between treatments by matching the spots in terms of their distribution, volume, relative intensity, pI, and MW. The spot data were tested for homogeneity of variances and normality using the Levene and Shapiro–Wilk tests, respectively. Subsequently, the differences in the means between treatments were analyzed using Student’s *t*-test. Additionally, the Mann–Whitney test (Wilcoxon rank-sum test) was used when the normality criteria were breached in any of the treatments. For both tests (Student or Mann–Whitney), significance was detected at the 0.05 level. For all data, trends were considered at 0.05 < *p* ≤ 0.10.

## 3. Results

### 3.1. Carcass and Meat Quality Traits

**Figure 1:**
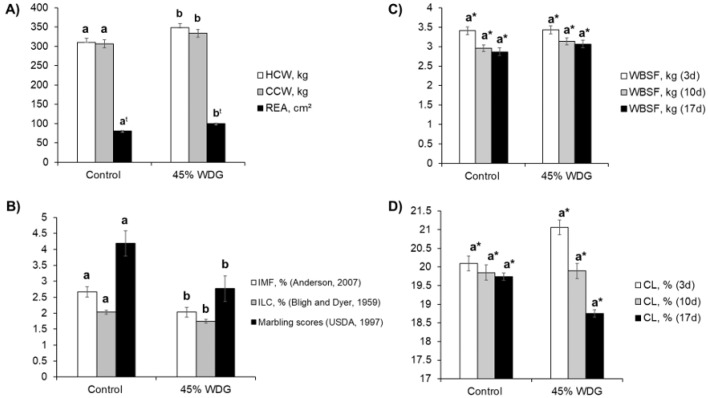
Carcass (**A**) and meat quality (**B**–**D**) traits of feedlot-finished F1 Angus-Nellore bulls fed on diets with 0% (control) and 45% corn wet distiller grains (WDG). HCW = hot carcass weight; CCW = cold carcass weight; REA = ribeye area; IMF = intramuscular fat; ILC = intramuscular lipid content; marbling score (0 = devoid, 2 = practically devoid, 3 = traces, 4 = slight, 5 = small, 6 = modest); WBSF = Warner–Bratzler shear force; CL = cooking loss. The bars indicate the standard deviation of the mean. ^a,b^ Different lowercase letters indicate differences at *p* < 0.05 except for REA (^t^), which has a *p*-value = 0.064 between feedlot diets. * Significant effects (*p* < 0.05) due to ageing time (3, 10, and 17 days post-mortem).

The feedlot-finished F1 Angus-Nellore bulls fed on diets with 45% WDG had a higher HCW and CCW (*p* < 0.05) compared to the control group (Figure 1A). The inclusion of WDG promoted an average increase of 10.5 and 10.25 kg in the HCW and CCW, respectively. In addition, there was a trend (*p* = 0.064) towards greater muscle growth, measured as REA, in the animals fed 45% WDG vs. the control diet (Figure 1A), complementing the HCW and CCW results. There were no differences (*p* > 0.10) in the BFT between the control vs. WDG diet (7.54 vs. 7.63 ± 0.56 mm).

Differences (*p* < 0.05) in the IMF content, ILC, and visual marbling scores were observed between the control and WDG treatment groups (Figure 1B). The deposition of IMF was reduced in the feedlot-finished animals fed on the diet with 45% WDG compared to the control treatment. There were no differences (*p* > 0.10) in the WBSF or CL evaluated at the three aging times (Figure 1C and Figure 1D, respectively). However, as expected, differences in the WBSF were observed between the three time points (3, 10, and 17 days post-mortem), regardless of the treatment.

**Figure 2:**
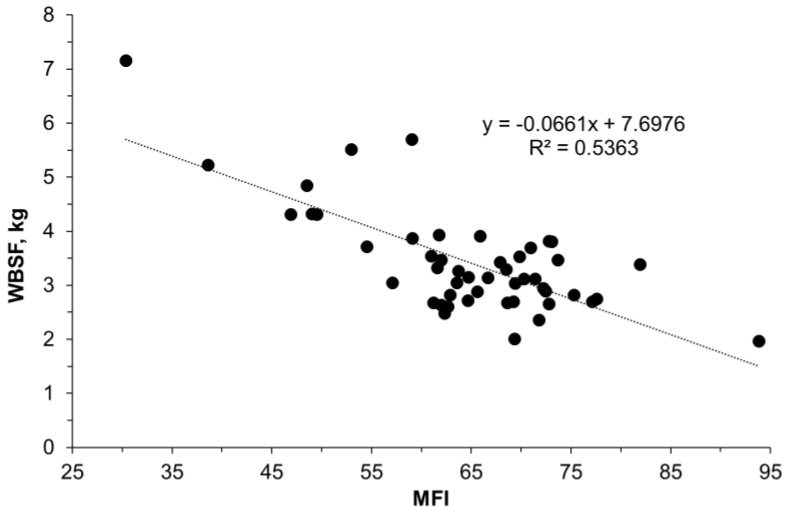
Relationship between meat tenderness, measured by the myofibril fragmentation index (MFI), and Warner–Bratzler shear force (WBSF) in the *Longissimus thoracis* muscle of feedlot-finished F1 Angus-Nellore bulls fed on the control diet or a diet with 45% wet distiller grains.

In the present study, the WBSF values were as expected, being inversely related to the MFI data (Figure 2). There were no differences between the control and WDG treatments (*p* > 0.05).

### 3.2. Fatty Acid Profile

To better understand the effects of the diet containing WDG on the lipid profile of meat, we determined the fatty acid profile of the main lipid sources used in the diets (control vs. 45% WDG), including soybean meal, dry ground corn, and WDG (Table 1).

The dietary inclusion of WDG reduced (*p* < 0.05) the IMF content of the meat compared to the control diet when the results were expressed as percentages (2.03 ± xx vs. 2.67 ± 0.16) or marbling scores (2.77 ± xx vs. 4.19 ± 0.4). The fatty acid composition of the LT muscle is presented in Table 2. The concentrations of C10:0, C11:0, lauric (C12:0), C13:0 iso, myristic (C14:0), pentadecanoic (C15:0), palmitoleic (C16:1 c9), margaric (C17:0), heptadecenoic acid (C17:1), stearic (C18:0), oleic (C18:1 c9), C18:1 c11, C18:12, C18:1 c15, α-linolenic (C18:3 n3), and C20:1 acid decreased in the meat of bulls fed on WDG. However, a decrease was observed in the total SFA (*p* < 0.01), UFA (*p* = 0.001) and MUFA (*p* = 0.001) concentrations of the LT muscle in the meat of bulls fed on WDG. In addition, there was a decrease in the MUFA:SFA (*p* = 0.001) and UFA:SFA (*p* = 0.004) ratios when WDG was included in the diet.

### 3.3. Muscle Tissue Proteome

**Figure 3:**
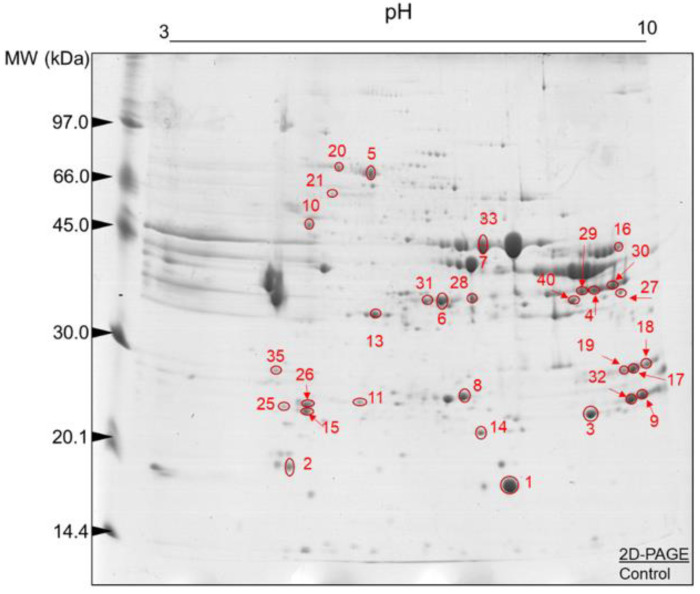
Protein spots selected for characterization by mass spectrometry (ESI-MS) after image analysis. Two-dimensional polyacrylamide gel electrophoresis (2D-PAGE): 12.5% (*w/v*) and pH gradient 3–10. Muscle tissue samples (*Longissimus thoracis*) from feedlot-finished F1 Angus-Nellore bulls fed on diets without the inclusion of wet corn distiller grains (control).

**Figure 4:**
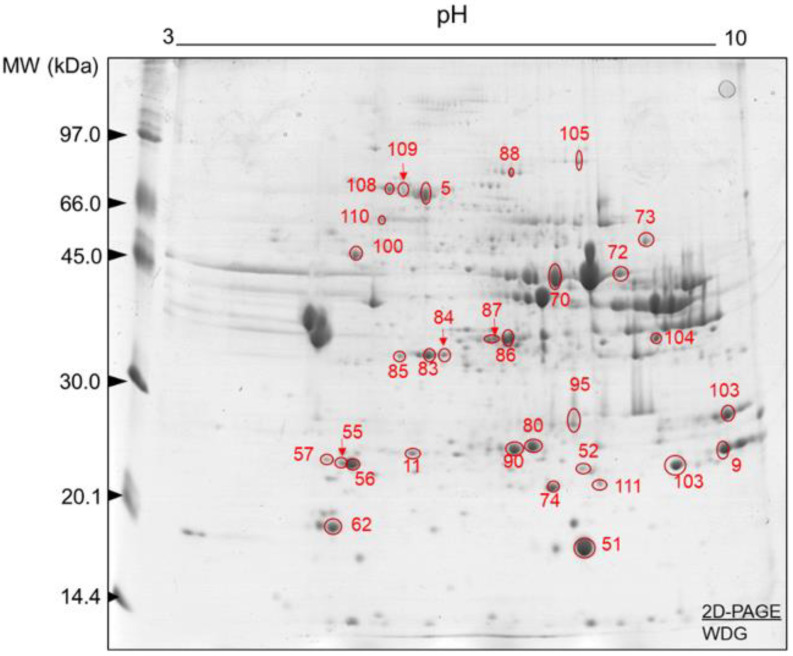
Protein spots selected for characterization by mass spectrometry (ESI-MS) after image analysis. Two-dimensional polyacrylamide gel electrophoresis (2D-PAGE): 12.5% (*w/v*) and pH gradient 3–10. Muscle tissue samples (*Longissimus thoracis*) from feedlot-finished F1 Angus-Nellore bulls fed on diets with the inclusion of wet corn distiller grains (WDG).

Representative gels obtained from LT muscle samples (sampled early post-mortem) of the control and WDG treatment groups are illustrated in Figure 3 and Figure 4, respectively. In both groups, the 2D-PAGE gels showed a good resolution (matching >50%), indicating efficient protein separation. A great array of protein spots was observed over the pH 3–10 gradient, promoting a good separation of the proteins in this pI range. Furthermore, most protein spots were found mainly in the MW range from 20 to 66 kDa, with most p*I*s in the range of 5.0 to 7.0, respectively. However, the presence of protein spots with a lower MW (<20 kDa) must be highlighted.

The analysis of the images of the 2D-PAGE runs of the control and WDG groups revealed correlations between the gels of each treatment (*n* = 10) of 63% and 65%, respectively. These results indicate that the protein spots were present in the biological replicates of these gels. The mean numbers of protein spots in the gel replicates were 167 ± 25 and 162.2 ± 15.5 in the control and WDG groups, respectively. Several protein spots were characterized as the most expressed in the control (Table 3) and WDG (Table 4) treatments and subsequently used to better understand the main molecular signatures and pathways.

The BPs, MF, and CC of the identified proteins differed between the control (Figure S1) and WDG treatment (Figure S2). Considering the distribution of the 20 top-level gene ontology terms, BPs were identified to a greater extent in the control treatment, particularly cellular (40), regulatory (27) and metabolic (22) processes. The same BPs were observed in smaller numbers in the WDG treatment (27, 21, and 22, respectively). The same trend was found for the number of MFs identified in the control treatment (45) vs. WDG (30), with a predominance of proteins whose MFs were related to molecule binding, catalytic activity, structural activity, and chaperones in both treatments. This is further in agreement with the classifications depicted in Table 3 and Table 4. On the other hand, the levels of CC identified in the two treatments were rather similar.

**Figure 5:**
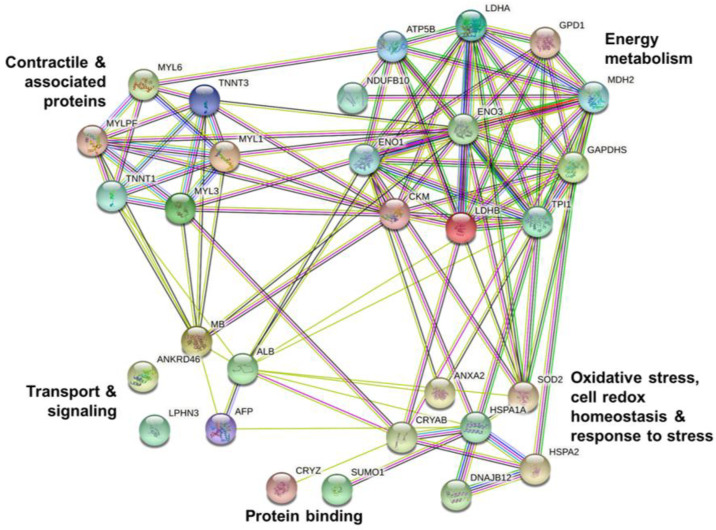
Analysis of protein–protein interactions using the differentially expressed proteins in the muscle tissue (*Longissimus thoracis*) of feedlot-finished F1 Angus-Nellore bulls fed on diets without the inclusion of wet corn distiller grains (control).

**Figure 6:**
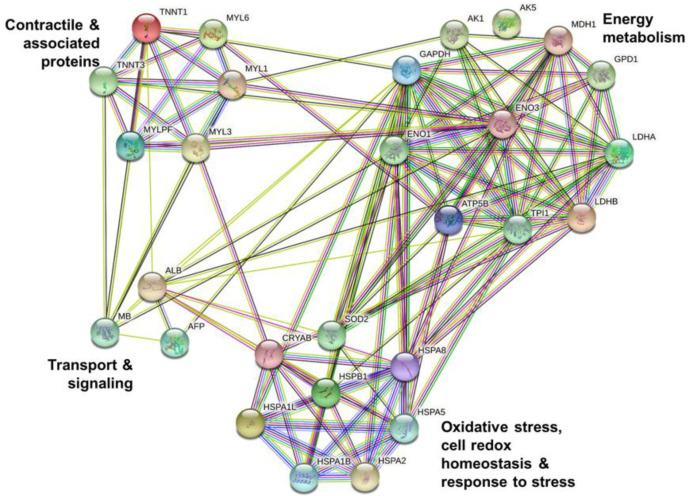
Analysis of protein–protein interactions using the differentially expressed proteins in the muscle tissue (*Longissimus thoracis*) of feedlot-finished F1 Angus-Nellore bulls fed on diets with the inclusion of 40% wet corn distiller grains (WDG).

The protein–protein interactions were analyzed in the control (Figure 5) and WDG (Figure 6) treatments.

These bioinformatic results demonstrated consistent interactions between the proteins characterized in the bovine muscle tissue samples. There were two main groups (clusters) of molecules in the control treatment: proteins related to energy metabolism (LDHA, LDHB, MDH2, GAPDHS, GPD1, NDUFB10, ATP5F1B, TPI1, ENO3, ENO1, and CKM) and proteins related to muscle contraction (MYLPF, MYL1, MYL3, MYL6, TNNT1, and TNNT3). Additionally, in the control treatment, a small interaction network involving proteins ATP5F1B (ATP synthase) and NDUFB10 (NADH dehydrogenase) was identified.

The same two groups (clusters) were also identified in the WDG treatment group, but these samples also included other proteins related to energy metabolism (LDHA LDHB, MDH1, GAPDH, GPD1, ATP5F1B, ENO3, ENO1, TPI1, TPI1, Ak5, and AK1). Protein AK5 (adenylate kinase isoenzyme 5) appears to have no interaction with any of the proteins identified in these animals. This finding may be related to the limited information available about the role of Ak5 in bovine muscle tissue.

**Figure 7:**
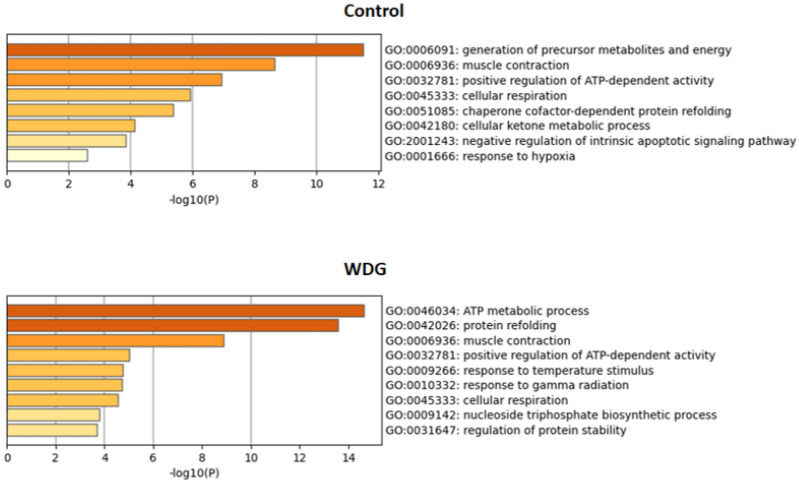
Enriched ontology clusters based on significantly enriched gene ontology (GO), obtained using the protein lists of the control (*n* = 30) and corn wet distiller grain (WDG) diet groups (*n* = 28) identified in the muscle tissue (*Longissimus thoracis*) of feedlot-finished F1 Angus-Nellore bulls. The graphs highlight all the enriched terms (functional clusters = 8 (control) and 9 (WDG)) of the protein list early post-mortem, highlighting the importance of the energy metabolism (metabolic and ATP process), muscle contraction, and apoptosis processes according to the Log *p*-values.

The main enriched terms and pathways identified in this study using the differentially expressed proteins for the control and WDG treatments are summarized in Figure 7, based on gene ontology terms. The energy metabolism pathway, through the “generation of precursor metabolites and energy”, was highly and significantly upregulated in the control compared to the WDG diet group. In animals fed on the WDG diet, cluster pathways related to the ATP metabolic process and protein refolding were more enriched, which helps us to explain the greater HCW and REA observed in these animals. Pathways of generation of metabolites and energy in the control diet group help us to explain the higher IMF found in the meat of these animals.

**Figure 8:**
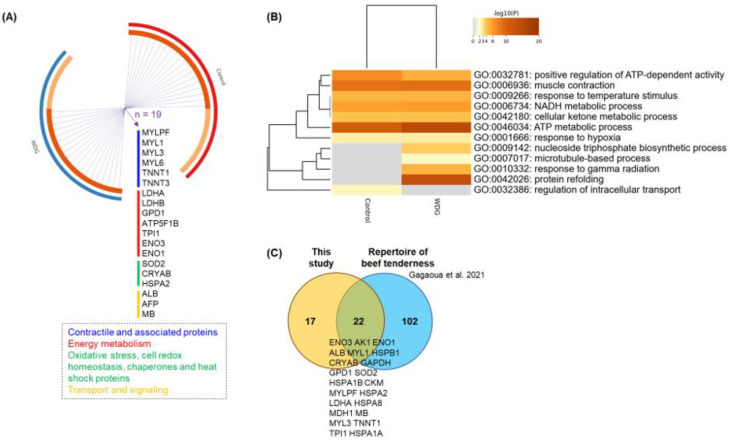
Gene ontology (GO) pathway and clustering. (**A**) Protein overlap analysis using a Circos plot, illustrating the degree of overlap between experimental diets (control vs. corn wet distiller grains—WDG) based on the list of 58 proteins identified in the muscle tissue (*Longissimus thoracis*) of feedlot-finished F1 Angus-Nellore bulls. Each outer arch represents a feedlot diet with a different color. On the inside, the dark orange color represents the proteins that appear in multiple lists and the light orange color represents proteins that are unique to that protein list. Purple lines link the same proteins (gene names) that are shared by the input. Nineteen proteins were found that were common to both treatments. (**B**) Hierarchical heatmap clustering indicating the first 12 enriched GO terms analyzed by Metascape^®^ (available online: https://metascape.org/, accessed on 1 August 2022), which were significant. The heatmap is colored, with the *p*-values indicated by colors, where grey cells indicate a lack of significant enrichment, pale brown indicates a low *p*-value, and dark brown indicates a high *p*-value. (**C**) Proteins described by Gagaoua et al. [31] using an integromic approach, in which 124 protein were biomarkers of beef tenderness. From this repertoire, 22 putative protein biomarkers of beef tenderness were also found to be impacted by the feeding regime in the current study (for protein details, see Table 3 and Table 4).

Further bioinformatic analyses allowed us to compare the protein overlap, using Circos plots, for the total number of 58 proteins between dietary treatments (control vs. WDG). Such an analysis displays the overlap and functional connections between genes and allowed us to compare the enriched ontology terms between the treatments in order to identify those that were common or specific to the treatments (Figure 8).

The current GO analysis (Figure 8B) suggests that the “nucleoside triphosphate biosynthetic process”, “response to gamma radiation”, “microtubule-based process”, and “protein refolding” are associated with, and specific to, the WDG treatment and can be associated with muscle growth (REA) and a greater hot carcass weight in the bulls fed on the WDG diet. Additionally, the “regulation of intracellular transport” was specifically associated with a greater IMF, found in animals fed on the control diet. Other GO terms were common to both protein lists, some of which were more significant for the WDG, such as “ATP metabolic process”.

Forty-one QTLs were identified for the carcass (*n* = 14) and meat quality traits (*n* = 27) on different chromosomes (Chr) (Table 5). The CRYAB on Chr.15 was common to two carcass traits, including the carcass weight and REA, while GPD1 on Chr.5 was related to the IMF content. Overall, fourteen chromosomes encompassed the 29 proteins (gene names), and among the major QTLs, most of the proteins were related to the “energy metabolism” pathway, followed by the “muscle contraction and structure” pathways and “apoptosis processes”. Several of the proteins found to change in this study were biomarkers of both beef tenderness and QTLs.

## 4. Discussion

Tenderness and juiciness are among the most important meat quality attributes for consumers in some markets, who are willing to pay more for higher-quality products [32]. Meat quality research using proteomic approaches can help us to identify the markers of higher-quality products by enabling the overall analysis of cellular proteins using biotechnologies, such as two-dimensional electrophoresis, mass spectrometry, and bioinformatics [12,25,33]. To the best of our knowledge, this is the first study that used a proteomic approach to decipher the biochemical and molecular mechanisms that regulate meat tenderness, marbling, and the fatty acid profile in feedlot-finished cattle fed on a diet containing WDG.

### 4.1. Carcass and Meat Quality Traits

The trend towards higher muscle growth, measured as REA, could be due to the higher carcass weights of the animals fed on 45% WDG compared to the control group. The REA reflects carcass muscularity. Consequently, heavier carcasses with a greater REA can lead to an increased cut yield in beef. The yield implies the direct financial return for the producer and slaughterhouse, since it is directly related to the amount of commercializable meat [34]. However, a higher carcass yield also depends on the BFT, a carcass trait that was similar between the two treatments in the present trial. Divergent results for the same carcass traits have been reported in the literature for feedlot-finished Hereford steers fed on diets containing 45% sorghum distiller grains [35], suggesting that the divergences between studies are due to the type of feed tested and the genotype.

Beef tenderness is affected by different factors, such as the genotype, nutrition, and forms of carcass and meat cut processing by the industry [36]. In the present study, WBSF values were not influenced by the inclusion of 45% WDG in the feedlot diet of crossbred bulls, with a difference only being observed between aging times. Similar results have been reported in other studies that found no effect of distiller grains on the objective tenderness [37] or sensory attributes [38].

Intramuscular fat, also known as marbling, is the most important fraction of adipose tissue, from a nutritional point of view, because it is present within the edible portion. This fat is found in several meat cuts and is closely associated with the main organoleptic characteristics of meat, conferring a very specific final flavor [39]. The reduction in starch levels in the diet with 45% WDG is probably the reason for the lower IMF content in the LT muscle of these animals. According to the literature, this dietary condition can lead to changes in IMF deposition [40]. With less substrate for IMF synthesis, changes in the gene expression and protein–protein interactions related to nutrient transport and energy metabolism occurred in the LT muscle of the animals fed on feedlot diets with 45% WDG.

The development of post-slaughter *rigor mortis* is one of the essential conditions for the onset of a subsequent event. The proteolysis of the structural components of sarcomeres, fostering the binding reactions of proteolytic enzymes to their cellular substrates, is an event that strongly depends on the development of *rigor mortis* and related pathways, such as apoptosis, identified in this study as an impacted process. In fact, apoptosis is an early event that occurs in the post-mortem muscle and is suggested to play a pivotal role in meat quality determination [10]. Enzymatic proteolysis involves a relatively large set of proteins, with the reactions being catalyzed by enzymes such as caspases, calpains, or cathepsins with a low or high affinity for their substrates. According to the literature, the MFI, which is a proxy allowing for the evaluation of post-mortem proteolysis, can explain more than 50% of the variation in beef tenderness and shows a high positive (r = 0.75) correlation with sensory tenderness and a negative (r = −0.72) correlation with WBSF values [20]. In the present study, although an inverse relationship was observed between these quality variables, neither myofibrillar proteolysis nor the WBSF was affected by the treatments, as described in earlier studies on Zebu cattle [21,41].

### 4.2. Fatty Acid Profile

The reduction in the IMF content of meat produced with the inclusion of 45% WDG may explain, in part, the alteration in the fatty acid profile when compared to the control treatment. The proteome of animals fed WDG exhibited a lower expression of proteins such as SOD2, TPI1, MDH2, GPD1, ENO3, ENO1, ALB, LDHA, LDHB, and GAPDHS, which may be related not only to the lower IMF content but also to the reduced expression of glycerol-3-phosphate dehydrogenase, an enzyme associated with the differentiation of adipocytes [42]. Together with the other proteins that were less expressed when WDG was included in the diet, these findings may explain the lower IMF content.

The addition of distiller grains to ruminant diets has been shown to be associated with a reduction in the endogenous synthesis of MUFA and increased incorporation of PUFA in meat [43]. Our results only confirm the reduction in the MUFA concentrations, since the inclusion of WDG did not alter the PUFA concentrations. The reduced endogenous synthesis of MUFA might be related to the lower activity of ∆9 desaturase, an enzyme that converts saturated fatty acids, such as stearic acid (C18:0), into oleic acid (C18:1 c9).

In this respect, we previously reported and observed that the inclusion of WDG in cattle diets reduced the expression of the SDC-1 gene [44], which encodes this enzyme. The lower synthesis of MUFA as a result of the lower expression of the SDC-1 gene was associated with the dietary inclusion of WDG. Moreover, this result may be associated with a reduction in starch consumption by the animals subjected to this treatment, since the expression of this gene is higher in animals fed on high-starch diets [45]. However, it should be noted that the reduction in MUFA did not compromise the meat fatty acid profile of the animals on the WDG treatment.

### 4.3. Muscle Proteome Data and Changing Molecular Signatures

The experimental diets affected the muscle tissue proteome of the cattle without altering meat traits such as the tenderness or color. Animals fed on 45% WDG exhibited a higher expression of proteins related to interconnected pathways, such as energy metabolism (LDHA LDHB, MDH1, GAPDH, GPD1, ATP5F1B, ENO3, ENO1, TPI1, Ak5, and AK1) and muscle contraction (MYL1, MYLPF, MYL1, MYL3, MYL6, TNNT1, and TNNT3). Some of the proteins identified have been related to muscle growth in previous studies [46,47] and may explain the trend towards an increase in the REA and HCW in the animals treated with 45% WDG compared to the control. In a study on sheep fed on feedlot diets containing cottonseed co-products [48], the authors found that the experimental diets promoted an increase in TNNT3 and MYL1 in the LT muscle, proteins responsible for the regulation of myosin. Thus, the diets affected the main factors involved in muscle contraction, as observed in the present study.

Other studies evaluated the meat quality of Nellore (*Bos indicus*) and Aberdeen Angus (*Bos taurus*) cattle using phosphoproteomics [49]. The authors described the presence of proteins involved in muscle fiber types (MYL1, MYL3, and MYL6) and energy metabolism (ENO3, ENO1, LDHA, LDHB, and GAPDHS) in these animals. These proteins were identified as potential biomarkers of muscle growth, meat tenderness, and IMF [31,33] (ref). In the present study, proteins such as SOD2, TPI, MDH1, GPD1, ENO3, ENO1, ALB, LDHA, LDHB, and GAPDHS were more expressed in the LT muscle of animals fed on the control diet. This finding may be related to the higher IMF content of the meat of these animals.

Among the several proteins that were more expressed in the control treatment, those related to energy metabolism (LDHA LDHB, MDH1, GAPDHS, GPD1, ATP5F1B, ENO3, ENO1, TPI1, TPI1, Ak5, and AK1) should be highlighted. The genes encoding these molecules have been described in other studies on beef cattle as biomarkers of carcass and meat quality traits such as the REA [50], IMF [51], tenderness [31], and color [52]. For example, HSPs possess anti-apoptotic properties and may contribute to delaying the onset of post-mortem apoptosis, thus influencing the development of tenderness and the conversion rate of muscle to meat [25,52]. However, there were no differences in meat tenderness between the control and 45% WDG groups. The protein biomarkers of beef tenderness identified in both treatments may help us to rule out possible differences in WBSF. Further investigations are needed in this context.

On the other hand, the greater abundance of GAPDHS in the control group may be related to lower insulin sensitivity, which helps to explain the lower IMF content observed in these animals. The GAPDHS is a key molecule in energy metabolism, including glucose transport, glycolysis, Krebs cycle, mitochondrial respiratory chain, and the ß-oxidation of fatty acids [11].

Some important factors must be considered when the protein expression in muscle tissue is correlated with meat quality traits, such as the end-point cooking temperature. Within this context, the study by Gagaoua et al. [53] analyzed meat samples from three breeds (Aberdeen Angus, Limousin and Blond d’Aquitaine) cooked at two end-point cooking temperatures (55 and 74 °C), and the sensory tenderness was evaluated by trained panelists in two different countries (France and the UK). The authors reported six proteins related to muscle structure and contraction (MYHC7, MYH2 and MYH1), oxidative stress (PARK7 and PRDX6), and proteolysis (CAPN1) that were affected regardless of the cooking temperature, country of origin of the panelists, or animal breed. These proteins can be considered as predictors of sensory meat tenderness. Thus, when proteins are used as predictors of meat quality, it is important to consider factors that modify the muscle tissue proteome [54,55], especially during the finishing period (initial live weight, duration, dietary concentrate level, consumption, among others), as well as carcass-associated factors (REA, BFT, and marbling), as recently discussed in several studies [12,56].

The inclusion of WDG (and consequent reduction in starch content) in the feedlot diet possibly resulted in changes in the ruminal metabolism that caused alterations in the protein expression in the LT muscle and, consequently, in the IMF deposition of these animals. Additionally, the reduction in starch content associated with the dietary inclusion of WDG preserved post-mortem proteolysis and, thus, did not compromise the meat tenderness.

Less IMF deposition was observed in the present study, which seems to reflect changes in the energy and lipid metabolism, indirectly measured by the analysis of the animals’ proteome levels. The replacement of corn/soybean meal ingredients with WDG alters dietary protein degradability. WDG is an ingredient rich in rumen undegradable protein, and researchers [5] have suggested that a greater protein supply to the intestine may stimulate the secretion of pancreatic amylase (increasing post-ruminal starch digestion). In the present study, the assessment of the physical, chemical, and molecular characteristics of the LT muscle permitted us to understand the alterations in IMF deposition in the animals. These results could be associated with the greater supply of rumen undegradable protein and glutamic acid due to the inclusion of WDG in the diet of these animals.

## 5. Conclusions

The inclusion of WDG in the feedlot diet of F1 Angus-Nellore bulls affected the protein expression, the protein–protein interactions in the LT muscle, and important metabolic pathways, including the ATP production, growth, and IMF deposition in these animals. However, there was no negative impact on post-mortem proteolysis, meat tenderness, or the fatty acid profile of the animals. This is the first study to describe proteome data for the muscle tissue of feedlot-finished beef cattle fed on high-WDG diets.

## Figures and Tables

**Table 1:** Dry matter (DM), ether extract (EE), and concentrations of the main fatty acids from the lipid sources used in the diets.

	Soybean Meal	Ground Corn	Corn Wet Distiller Grain
DM (% as fed)	89	88	32
EE (% DM)	2.0	4.3	4.0
Fatty acids (% of total fatty acids) ^a^			
Myristic C14:0	0.13	0.06	1.48
Palmitic C16:0	20.43	16.53	14.84
Stearic C18:0	4.29	1.99	9.73
Oleic C18:1 c9	14.85	28.30	23.1
Linoleic C18:2 c9-c12	50.77	49.27	2.48
Linolenic C18:3 n3	6.17	1.22	0.78
ΣSFA	26.07	19.55	27.35
ΣUFA	73.79	80.46	33.26
ΣMUFA	16.85	29.97	30.32
ΣPUFA	56.95	50.49	2.94

^a^ SFA = saturated fatty acids; UFA = unsaturated fatty acids; MUFA = monounsaturated fatty acids; PUFA = polyunsaturated fatty acids; ΣSFA = sum of the saturated fatty acids; sum of the unsaturated fatty acids; ΣMUFA = sum of the monounsaturated fatty acids; ΣPUFA = sum of the polyunsaturated fatty acids.

**Table 2:** Effects of wet corn distiller grains (WDG) on the percentages of the main fatty acids in the intramuscular fat of longissimus muscle of F1 Angus-Nellore bulls.

	Control	45% WDG	SEM	*p*-Value
Intramuscular fat, %	2.90	1.85	0.095	<0.0001
Fatty acids (mg/100 g of meat) ^a^				
C6:0	1.849	1.075	0.383	0.077
C10:0	1.829	1.149	0.219	0.037
C11:0	0.099	0.033	0.012	0.003
Lauric C12:0	2.343	1.127	0.240	0.009
C13:0 iso	0.341	0.103	0.055	0.001
C14:0 iso	0.717	0.503	0.185	0.861
Myristic C14:0	87.829	51.924	7.358	0.009
C15:0 iso	1.897	1.550	0.283	0.410
C15:0 anteiso	4.375	3.454	0.694	0.487
Myristoleic C14:1 c9	18.741	10.337	2.568	0.066
Pentadecanoic C15:0	16.069	6.996	1.177	<0.0001
C16:0 iso	1.795	1.278	0.367	0.492
Palmitic C16:0	733.76	468.36	24.679	0.347
C17:0 iso	5.196	4.753	0.526	0.567
Palmitoleic C16:1 c9	105.11	52.987	7.378	0.001
Margaric C17:0	34.703	16.794	1.807	0.001
Heptadecenoic C17:1	29.482	11.380	2.728	<0.0001
Stearic C18:0	378.11	262.61	17.397	0.002
C18:1 trans	75.933	75.957	11.334	0.998
Oleic C18:1 c9	1062.82	645.70	61.888	0.001
C18:1 c11	78.606	40.235	5.234	0.001
C18:1 c12	8.163	5.821	0.391	0.003
C18:1 c13	11.839	5.478	2.013	0.104
C18:1 t16	1.855	2.101	0.427	0.694
C18:1 c15	1.734	1.134	0.166	0.034
Linoleic C18:2 c9c12	131.04	99.794	18.983	0.278
C20:0	2.082	1.586	0.203	0.077
C18:3 n6	0.851	0.532	0.205	0.303
α-Linolenic C18:3 n3	10.389	6.348	0.882	0.012
C20:1	3.017	1.002	0.413	0.009
CLA C18:2 c9t11	3.877	4.772	0.826	0.465
C18:2 t10c12	0.094	0.201	0.075	0.607
C20:2	1.306	0.921	0.219	0.248
C20:3 n6	5.702	4.974	1.185	0.676
C22:0	0.0686	0.125	0.029	0.676
Arachidonic C20:4 n6	32.616	24.869	6.922	0.452
EPA C20:5 n3	7.576	5.204	1.434	0.276
C24:0	0.076	0.0552	0.021	0.505
C24:1	2.855	1.982	0.616	0.346
C22:5	14.983	10.644	2.806	0.306
C22:6 n3	1.500	1.273	0.325	0.634
ΣSFA	1271.29	822.41	34.943	<0.0001
ΣUFA	1610.15	1013.69	83.816	0.001
ΣMUFA	1400.21	854.16	76.630	0.001
ΣPUFA	209.94	159.53	31.540	0.291
ΣMUFA/ΣSFA	32.418	19.179	2.519	0.001
ΣPUFA/ΣSFA	4.887	3.615	0.776	0.280
ΣUFA/ΣSFA	37.305	22.794	2.854	0.004
Σn3	19.465	12.826	2.579	0.106
Σn6	39.169	30.376	8.284	0.474
ω-6/ω-3	54.802	42.263	6.873	0.233

^a^ SFA = saturated fatty acids; UFA = unsaturated fatty acids; MUFA = monounsaturated fatty acids; PUFA = polyunsaturated fatty acids. ΣSFA = sum of the saturated fatty acids; sum of the unsaturated fatty acids; ΣMUFA = sum of the monounsaturated fatty acids; ΣPUFA = sum of the polyunsaturated fatty acids.

**Table 3:** Proteins identified by mass spectrometry in *Longissimus thoracis (*LT) muscle of F1 Angus-Nellore bulls fed on the control diet.

Spot ID	Uniprot ID	Gene Symbol	Full Protein Names	Mascot Score	Protein Coverage (%)	pI/MW Experimental	pI/MW Theoretical
*Contractile and associated proteins*							
02	Q0P571	MYLPF	Myosin regulatory light chain 2, skeletal muscle isoform	35,055.73	53.53	3.69/19,127.56	4.735/19,012.55
15	A0JNJ5	MYL1	Myosin light chain 1/3, skeletal muscle isoform	6287.59	63.54	3.78/21,045.95	4.82/20,931.84
25	P85100	MYL3	Myosin light chain 3	1868.16	32.16	3.6/22,110.17	4.87/21,939.03
26	P60661	MYL6	Myosin light polypeptide 6	354.34	10.6	4.44/17,101.18	4.41/16,930.05
06	Q8MKH6	TNNT1	Troponin T, slow skeletal muscle	208.97	4.56	3.79/31,284.30	5.67/31,284.25
04	Q8MKI3	TNNT3	Troponin T, fast skeletal muscle	22,402.47	19.93	9.59/321,259.78	6.07/32,125.94
*Energy metabolism*							
40	P19858	LDHA	L-lactate dehydrogenase A chain	69.01	5.12	9.97/36,939.87	7.85/36,597.64
28	Q5E9B1	LDHB	L-lactate dehydrogenase B chain	51.97	6.59	6.56/37,009.55	6.13/36,723.64
29	Q32LG3	MDH2	Malate dehydrogenase, mitochondrial	257.13	18.05	9.73/36,124.77	8.45/35,668.5
30	Q2KJE5	GAPDHS	Glyceraldehyde-3-phosphate dehydrogenase, testis-specific	969.47	6.08	8.29/43,687.24	7.98/43,287.96
31	Q5EA88	GPD1	Glycerol-3-phosphate dehydrogenase [NAD(+)], cytoplasmic	51,064.30	65.90	4.26/38,218.10	6.54/37,647.67
32	Q02373	NDUFB10	NADH dehydrogenase [ubiquinone] 1 beta subcomplex subunit 10	2416.24	27.27	6.95/21,250.06	8.41/20,964.84
10	P00829	ATP5F1B	ATP synthase subunit beta, mitochondrial	125,429.60	56.44	5.32/56,284.23	5.04/56,283.53
08	Q5E956	TPI1	Triosephosphate isomerase	408,896.4	93.98	5.31/26,917.47	6.59/26,689.51
07	Q3ZC09	ENO3	Beta-enolase	250,468.30	69.59	4.26/47,438.48	7.55/47,096.01
33	Q9XSJ4	ENO1	Alpha-enolase	7368.94	34.56	4.26/47,668.70	6.48/47,326.13
16	Q9XSC6	CKM	Creatine kinase M-type	81.84	7.87	9.73/4322.14	6.80/42,988.95
*Oxidative stress, cell redox homeostasis, chaperones and heat shock proteins*							
14	P41976	SOD2	Superoxide dismutase [Mn], mitochondrial	4030.24	28.38	4.44/24,890.60	8.53/24,637.95
20	Q27975	HSPA1A	Heat shock 70 kDa protein 1A	72,555.56	54.45	4.07/70,544.33	5.64/70,258.51
21	P34933	HSPA2	Heat shock-related 70 kDa protein 2	504.82	5.19	4.07/70,024.93	5.32/69,739.7
09	Q58DR2	DNAJB12	DnaJ homolog subfamily B member 12	58.07	8.65	4.26/41,568.48	8.69/41,340.28
14	P02510	CRYAB	Alpha-crystallin B chain	16,025.71	73.14	5.32/20,036.81	7.05/20,036.79
06	P04272	ANXA2	Annexin A2	136.22	12.68	5.23/38,897.28	7.03/38,612.07
*Protein binding*							
17	Q5E9D1	SUMO1	Small ubiquitin-related modifier 1	141.4561	30.69	3.93/11,614.06	5.27/17,949.28
27	O97764	CRYZ	Zeta-crystallin	1171.379	37.88	9.26/35,553.94	8.15/35,382.8
*Transport and signaling*							
18	O97827	ADGRL3	Adhesion G protein-coupled receptor L3	58.17	3.04	6.56/67,847.39	7.82/67,329.78
35	Q3SX00	ANKRD46	Ankyrin repeat domain-containing protein 46	73.30	13.16	4.17/25,557.93	5.44/25,329.79
05	P02769	ALB	Albumin	14,091.32	78.25	4.26/71,289.43	5.87/69,293.41
19	Q3SZ57	AFP	Alpha-fetoprotein	137.99	1.15	6.64/70,412.61	5.98/68,587.64
01	P02192	MB	Myoglobin	373,870.70	87.66	9.98/170,776.21	7.19/17,077.59

**Table 4:** Proteins identified by mass spectrometry in *Longissimus thoracis* (LT) muscle of F1 Angus-Nellore bulls fed on a diet with 45% corn wet distiller grains.

Spot ID	Uniprot ID	Gene Symbol	Full Protein Names	Mascot Score	Protein Coverage (%)	pI/MW Experimental	pI/MW Theoretical
*Contractile and associated proteins*							
52	A0JNJ5	MYL1	Myosin light chain 1/3, skeletal muscle isoform	68,715.63	63.54	6.87/20,856.32	4.83/20,931.84
62	Q0P571	MYLPF	Myosin regulatory light chain 2, skeletal muscle isoform	74,702.68	63.53	3.82/19,126.56	4.73/19,012.55
55	A0JNJ5	MYL1	Myosin light chain 1/3, skeletal muscle isoform	68,715.63	63.54	7.00/21,459.50	4.83/20,931.84
56	P85100	MYL3	Myosin light chain 3	1554.92	12.56	7.6/22,110.17	4.87/21,939.03
57	P60661	MYL6	Myosin light polypeptide 6	316.95	10.6	4.44/17,101.18	7.86/52,285.46
72	Q8MKH6	TNNT1	Troponin T, slow skeletal muscle	1464.83	14.45	9.77/31,284.03	5.67/31,284.25
73	Q8MKI3	TNNT3	Troponin T, fast skeletal muscle	13,253.61	19.93	9.59/32,126.78	6.07/32,125.94
*Energy metabolism*							
83	P19858	LDHA	L-lactate dehydrogenase A chain	4527.98	29.22	6.10/36,939.71	7.85/36,597.64
14	Q5E9B1	LDHB	L-lactate dehydrogenase B chain	272.92	17.66	6.1/37,008.86	6.13/36,723.64
85	Q3T145	MDH1	Malate dehydrogenase, cytoplasmic	346.09	20.36	4.07/36,723.40	6.25/36,438.19
86	P10096	GAPDH	Glyceraldehyde-3-phosphate dehydrogenase	59.52	8.71	6.1/36,096.27	8.29/35,868.09
87	Q5EA88	GPD1	Glycerol-3-phosphate dehydrogenase [NAD(+)]	239.72	23.78	4.26/38,218.01	6.54/37,647.67
100	P00829	ATP5F1B	ATP synthase subunit beta, mitochondrial	42,545.85	39.77	6.78/56,283.25	5.04/56,283.53
70	Q3ZC09	ENO3	Beta-enolase	6853.10	48.62	6.29/47,438.25	7.55/47,096.01
70	Q9XSJ4	ENO1	Alpha-enolase	3591.51	17.74	6.29/47,668.37	6.48/47,326.13
80	Q5E956	TPI1	Triosephosphate isomerase	367,356.90	93.57	6.89/26,917.47	6.59/26,689.51
90	Q5E956	TPI1	Triosephosphate isomerase	1955.57	32.13	5.23/26,917.64	6.59/26,689.51
104	A4IFD0	Ak5	Adenylate kinase isoenzyme 5	6634.23	1.60	6.78/63,843.41	4.91/63,272.94
103	P00570	AK1	Adenylate kinase isoenzyme 1	8082.40	48.45	7.53/21,778.04	8.35/21,663.94
*Oxidative stress, cell redox homeostasis, chaperones and heat shock proteins*							
95	P41976	SOD2	Superoxide dismutase [Mn], mitochondrial	4030.24	28.38	7.00/24,809.60	8.53/24,637.95
08	Q27965	HSPA1B	Heat shock 70 kDa protein 1B	642.10	27.15	4.07/70,513.42	5.64/70,228.42
108	P19120	HSPA8	Heat shock cognate 71 kDa protein	107.21	3.69	4.07/71,468.73	5.25/71,240.51
109	P0CB32	HSPA1L	Heat shock 70 kDa protein 1-like	188.49	9.83	3.96/70,788.37	5.91/70,389.07
110	P34933	HSPA2	Heat shock-related 70 kDa protein 2	107.21	3.77	4.07/70,024.93	5.32/69,739.7
111	Q3T149	HSPB1	Heat shock protein beta-1	158,366.90	86.57	6.78/22,450.99	6.11/22,393.06
74	P02510	CRYAB	Alpha-crystallin B chain	90,018.13	70.86	5.32/20,036.06	7.05/20,036.79
88	Q0VCX2	HSPA5	Endoplasmic reticulum chaperone BiP	79.91	1.68	4.07/72,514.18	4.93/72,400.03
*Transport and signaling*							
05	P02769	ALB	Albumin	30,645.09	50.74	4.26/71,289.30	5.87/69,293.41
105	Q3SZ57	AFP	Alpha-fetoprotein	42.61	1.15	6.64/70,410.61	5.98/68,587.64
51	P02192	MB	Myoglobin	1483.74	16.88	4.42/17,077.21	7.20/17,077.59

**Table 5:** List of the quantitative trait loci (QTL) of the carcass and meat quality traits and their chromosomes (Chr.) obtained using the list of the 58 proteins from the LT muscle of F1 Angus-Nellore bulls fed on the control vs. corn wet distiller grain diet (WDG).

QTL Linked to QTLdb ^a^	Gene Name	Protein Name	UniProtID (Bovine)	Chr.
Carcass weight (*n* = 10)	MDH1	Malate dehydrogenase, cytoplasmic	Q3T145	Chr.11
	CRYAB	Alpha-crystallin B chain	P02510 V6F832	Chr.15
	ENO1	Alpha-enolase	Q9XSJ4	Chr.16
	MYLPF	Myosin regulatory light chain 2	Q0P571	Chr.25
	MDH2	Malate dehydrogenase, mitochondrial	Q58DR9 Q32LG3	Chr.25
	HSPB1	Heat shock protein beta-1	Q3T149 E9RHW1	Chr.25
	LDHA	L-lactate dehydrogenase A chain	P19858	Chr.29
	AK5	Adenylate kinase isoenzyme 5	A4IFD0	Chr.3
	CRYZ	Zeta-crystallin	O97764	Chr.3
	MB	Myoglobin	A0A1K0FUF3 P02192	Chr.5
Ribeye area (*n* = 3)	CRYAB	Alpha-crystallin B chain	P02510 V6F832	Chr.15
	MB	Myoglobin	A0A1K0FUF3 P02192	Chr.5
	LDHB	L-lactate dehydrogenase B chain	Q5E9B1	Chr.5
Marbling score (*n* = 6)	HSPA8	Heat shock cognate 71 kDa protein	P19120	Chr.15
	SUMO1	Small ubiquitin-related modifier 1	Q5E9D1	Chr.2
	HSPA1B HSPA1A	Heat shock 70 kDa protein 1B; Heat shock 70 kDa protein 1A	Q27965 Q27975	Chr.23
	HSPA1L	Heat shock 70 kDa protein 1-like	P0CB32	Chr.23
	Ak5	Adenylate kinase isoenzyme 5	A4IFD0	Chr.3
	CRYZ	Zeta-crystallin	O97764	Chr.3
Intramuscular fat (*n* = 1)	GPD1	Glycerol-3-phosphate dehydrogenase	Q5EA88	Chr.5
Oleic acid content (*n* = 1)	ENO3	Beta-enolase	Q3ZC09	Chr.19
Omega-3 unsaturated fatty acid content (*n* = 1)	ANXA2	Annexin A2	P04272	Chr.10
Palmitic acid content (*n* = 2)	GAPDHS	Glyceraldehyde-3-phosphate dehydrogenase, testis-specific	Q2KJE5	Chr.18
	CKM	Creatine kinase M-type	Q9XSC6	Chr.18
Palmitoleic acid content (*n* = 2)	HSPA2	Heat shock-related 70 kDa protein 2	P34933	Chr.10
	ENO3	Beta-enolase	Q3ZC09	Chr.19
Palmitoleic acid to palmitic acid ratio (*n* = 1)	DNAJB12	DnaJ homolog subfamily B member 12	Q58DR2	Chr.28
Stearic acid content (*n* = 4)	HSPA2	Heat shock-related 70 kDa protein 2	HSPA2	Chr.10
	MDH1	Malate dehydrogenase, cytoplasmic	MDH1	Chr.11
	HSPA5	Endoplasmic reticulum chaperone BiP	HSPA5	Chr.11
	AK1	Adenylate kinase isoenzyme 1	AK1	Chr.11
Trans-6/9-C18:1 fatty acid content (*n* = 1)	ATP5F1B	ATP synthase subunit beta, mitochondrial	ATP5F1B	Chr.2
Shear force (*n* = 8)	HSPA8	Heat shock cognate 71 kDa protein	P19120	Chr.15
	NDUFB10	NADH dehydrogenase	M5FHL5 Q02373	Chr.25
	MYLPF	Myosin regulatory light chain 2	Q0P571	Chr.25
	ADGRL3	Adhesion G protein-coupled receptor L3	O97827	Chr.6
	ALB	Albumin	P02769 A0A140T897	Chr.6
	ADGRL3	Adhesion G protein-coupled receptor L3	O97827	Chr.6
	ALB	Albumin	P02769 A0A140T897	Chr.6
	AFP	Alpha-fetoprotein	Q3SZ57	Chr.6

^a^ ProteQTL tool included in ProteINSIDE (available online: http://www.proteinside.org/, accessed on 1 August 2022) interrogates a public library of published QTL in the Animal QTL Database (available online: https://www.animalgenome.org/QTLdb/, accessed on 1 August 2022) that contains cattle QTL and association data curated from published scientific articles.

## Data Availability

All relevant data are within the paper.

## Supplementary Materials

The following supporting information can be downloaded at: https://www.mdpi.com/article/10.3390/foods11203233/s1. Table S1: Composition of the experimental diets. Figure S1: Classification of proteins identified in the muscle tissue (*Longissimus thoracis*) of feedlot-finished F1 Angus-Nellore bulls fed on diets without the inclusion of wet corn distiller grains (control). Proteins were separated by 2D-PAGE and identified by mass spectrometry (ESI-MS/MS). The OMICSBOX software was used to classify the proteins according to the biological process (BP), molecular function (MF), and cellular component (CC). Figure S2: Classification of proteins identified in the muscle tissue (*Longissimus thoracis*) of feedlot-finished F1 Angus-Nellore bulls fed on diets with the inclusion of wet corn distiller grains (WDG). Proteins were separated by 2D-PAGE and identified by mass spectrometry (ESI-MS/MS). The OMICSBOX software was used to classify the proteins according to the biological process (BP), molecular function (MF), and cellular component (CC).
