# Supplementary material for: Meat Quality and Muscle Tissue Proteome of Crossbred Bulls Finished under Feedlot Using Wet Distiller Grains By-Product
Source: Foods. 2022 Oct 17;11(20):3233. doi: 10.3390/foods11203233 (PMC9602256; doi:10.3390/foods11203233)
Supplement: Supplementary file 1 [file foods-11-03233-s001.zip › foods-1934084-supplementary.pdf]

Article

Meat Quality and Muscle Tissue Proteome of Crossbred Bulls Finished under Feedlot Using Wet Distiller Grains By-product

Welder Baldassini [1,4,*], Mohammed Gagaoua [2,3,*], Bismarck Santiago [4], Leone Rocha [1], Juliana Torrecilhas [1], Rodrigo Torres [1], Rogério Curi [1,4], Otávio Machado Neto [1,4], Pedro Padilha [5], Felipe Santos [6], Dante Pazzanese Lanna [7] and Luis Artur Chardulo [1,4]

[1] School of Veterinary Medicine and Animal Science (FMVZ), São Paulo State University (UNESP), Botucatu 18618-681, São Paulo, Brazil
[2] School of Agriculture and Veterinary Sciences (FCAV), São Paulo State University (UNESP), Jaboticabal 14884-900, São Paulo, Brazil
[3] Food Quality and Sensory Science Department, Teagasc Food Research Centre, Ashtown, Dublin 15, D15 DY05 Dublin, Ireland
[4] PEGASE, INRAE, Institut Agro, 35590 Saint-Gilles, France
[5] Institute of Bioscience (IB), São Paulo State University (UNESP), 18618-689 Botucatu, São Paulo, Brazil
[6] College of Sciences and Engineering, São Paulo State University (UNESP), Tupã 17602-496, São Paulo, Brazil
[7] "Luiz de Queiroz" College of Agriculture (ESALQ), University of São Paulo (USP), Piracicaba 13418-900, São Paulo, Brazil
* Correspondence: w.baldassini@unesp.br (W.B.); mohammed.gagaoua@inrae.fr or gmber2001@yahoo.fr (M.G.)

Supplementary Data

Table S1. Composition of the experimental diets.

Item	Control	WDG
Ingredients (g/kg of dry matter – DM)		
Tifton 85	42	42
Sugarcane bagasse	71	71
Ground corn	749.2	387.3
Soybean meal	103.6	11.0
Low fat corn wet distillers grains (WDG)	0	450
Mineral-vitamin supplement [a]	34.2	34.2
Potassium chloride	0	4.5
Nutritional Composition (g/kg of DM)		
Dry Matter (g/kg as fed)	867.8	607.2
Crude Protein	128.5	200.7
Ether Extract	34.2	35.5
Neutral Detergent Fiber	162.8	387.5
Starch	482.2	246.9
Ca: P ratio	2.22	1.33

[a] Mineral-vitamin supplement containing: 19.5% Ca; 1.9% S; 1.5% Mg; 4.5% Na; 1.6% P; 1715 ppm Zn; 1285 ppm Mn; 428 ppm Cu; 25 ppm I; 5.7 ppm Se; 8.5 ppm Co; 286 ppm Fe; 86000 UI Vit A; 115000 UI Vit D3; 128000 UI Vit E; 0.39% Urea; 945 ppm of sodium monensin.

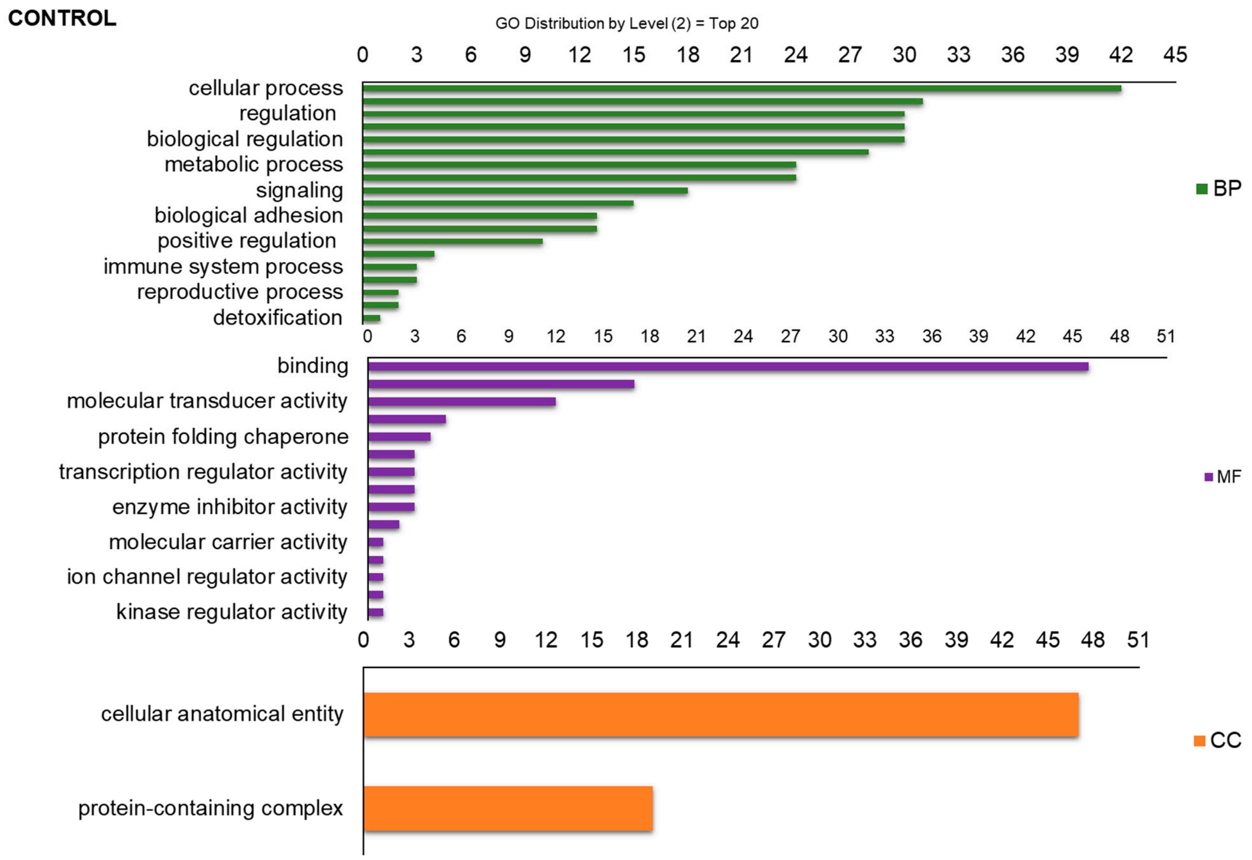

Figure S1. Classification of proteins identified in muscle tissue (*Longissimus thoracis*) of feedlot-finished F1 Angus-Nellore bulls fed diets without inclusion of wet corn distillers grains (control). Proteins were separated by 2D-PAGE and identified by mass spectrometry (ESI-MS/MS). The OMICSBOX software was used to classify the proteins according to biological process (BP), molecular function (MF), and cellular component (CC).

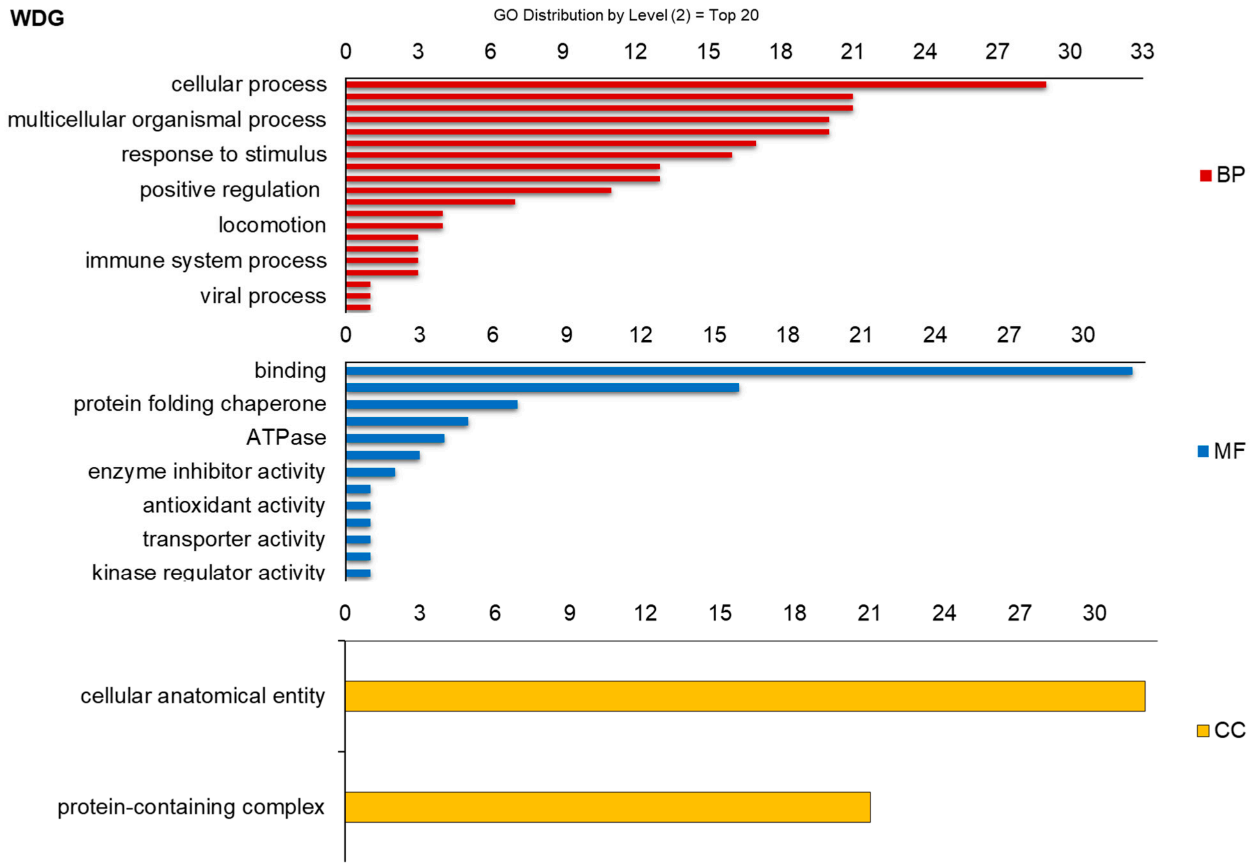

Figure S2. Classification of proteins identified in muscle tissue (*Longissimus thoracis*) of feedlot-finished F1 Angus-Nellore bulls fed diets with inclusion of wet corn distillers grains (WDG). Proteins were separated by 2D-PAGE and identified by mass spectrometry (ESI-MS/MS). The OMICSBOX software was used to classify the proteins according to biological process (BP), molecular function (MF), and cellular component (CC).
